# It Was Me: The Use of Sense of Agency Cues Differs Between Cultures

**DOI:** 10.3389/fpsyg.2019.00650

**Published:** 2019-03-22

**Authors:** Victoria K. E. Bart, Erdenechimeg Sharavdorj, Khishignyam Bazarvaani, Tegshbuyan Munkhbat, Dorit Wenke, Martina Rieger

**Affiliations:** ^1^Institute of Psychology, Department for Psychology and Medical Sciences, Private University for Health Sciences, Medical Informatics and Technology, Hall in Tirol, Austria; ^2^School of Arts and Sciences, Division of Social Sciences, Department of Education and Psychology, National University of Mongolia, Ulaanbaatar, Mongolia; ^3^School of Economics and Statistics, Guangzhou University, Guangzhou, China; ^4^Department of Psychology, PFH Private University of Applied Sciences, Göttingen, Germany

**Keywords:** sense of agency, affective valence, congruency, agency cues, cultural differences

## Abstract

Sense of agency (SoA) is the sense of having control over one’s own actions and through them events in the outside world. SoA may be estimated by integrating different agency cues. In the present study, we examined whether the use of different agency cues – action-effect congruency, temporal relation between action and effect, and affective valence of effects – differs between Eastern (Mongolian) and Western (Austrian) cultures. In a learning phase, participants learned to associate different actions (keypresses) with positive and negative action effects (smileys). In a test phase, participants performed the same keypresses. After different intervals positive and negative action effects, which were either congruent or incongruent with the previously acquired action-effect associations, were presented. In each trial participants were asked to rate how likely the action effect was caused by themselves or by the computer (authorship ratings). In both groups authorship ratings were higher for congruent compared to incongruent action effects and for positive compared to negative action effects. This indicates that action-effect congruency and affective valence of action effects modulate SoA. Further, in both groups the difference between positive and negative effects was higher with congruent effects than incongruent effects. This overadditive effect of action-effect congruency and affective valence might indicate that an integration of different agency cues takes place. Decreasing authorship ratings with increasing interval were observed in Austrians but not in Mongolians. For Mongolians, the temporal chronology of events might be less important when inferring causality. Therefore, information regarding the temporal occurrence of the effect might not be used as an agency cue in Mongolians. In conclusion, some agency cues might be similarly used in different cultures, but the use of others might be culture-dependent.

## Introduction

Sense of agency (SoA) is the sense of having control over one’s own actions and through them events in the outside world (Haggard and Tsakiris, 2009). It forms the basis for beliefs in free will (Aarts and van den Bos, 2011) and serves central social functions, like attribution of social or legal responsibility, which is vital to a functioning society (Haggard and Tsakiris, 2009; Moore, 2016; Haggard, 2017). Even though SoA is a core feature of human life, cultural differences in SoA might exist. Eastern and Western cultures differ in a variety of self-related psychological constructs like collectivism vs. individualism, self-critical vs. self-enhancing motivation, and the relation between self and others (Heine, 2001). Thus, one may expect that those cultural differences also extend to SoA (Frith, 2013). In the present study, we therefore investigated to what extent SoA differs between Eastern and Western cultures. In particular, we were interested in the question whether agency cues like the congruency between action and effect, the temporal relation between action and effect, and the affective valence of the effect influence SoA differently in Eastern (Mongolia) and Western (Austria) cultures.

Sense of agency can be assessed using indirect and direct measures. Indirect measures rely on perceptual differences between conditions evoking more and less SoA (Dewey and Knoblich, 2014). One such indirect measure is intentional binding. Intentional binding has been originally observed as a temporal illusion, which consists in the attraction of voluntary actions and effects toward one another compared to when either event occurs in isolation (Haggard et al., 2002). It has been suggested that intentional binding is stronger in conditions in which more SoA is experienced than in conditions in which less SoA is experienced (for a review see Moore and Obhi, 2012). In contrast, direct measures rely on direct judgments of SoA, for instance via rating scales (Dewey and Knoblich, 2014). In the present study, we used direct SoA judgments (authorship ratings).

According to cue integration models of SoA, SoA may be estimated by weighing various possible explanations for actions and their effects (Synofzik et al., 2008, 2009). An integration of multiple agency cues and their relative reliability in a given situation is necessary to obtain a valid estimate of SoA, i.e., to determine whether oneself or someone else is responsible for a certain action and/or effect (Synofzik et al., 2009; Moore and Fletcher, 2012).

A variety of different agency cues have been investigated. When an action is performed sensory consequences of the action are predicted (Wolpert, 1997; Wolpert and Flanagan, 2001). SoA occurs if internally predicted and actual sensory consequences match, whereas discrepancies between predicted and actual consequences weaken SoA (Frith et al., 2000; Blakemore et al., 2002). Thus, actions like pushing or pulling and the experience of effects which typically result from those actions (i.e., objects moving away or coming closer) enhance intentional binding and SoA ratings in comparison to situations in which untypical effects occur (Ebert and Wegner, 2010). Similarly, SoA ratings are higher in conditions in which the effect of an action is congruent with previously acquired action-effect associations compared to when it is incongruent (Sato and Yasuda, 2005; Spengler et al., 2009). Moreover, the temporal relation between action and effect modulates SoA (Berberian et al., 2012; Haering and Kiesel, 2015). Based on previous experiences of usual action-effect intervals, predictions about the timing of a certain effect in relation to the action that produces it are formed (Haering and Kiesel, 2012; Walsh and Haggard, 2013). SoA ratings are higher when the temporal prediction of the effect matches the actual timing (Haering and Kiesel, 2015). In addition to temporal prediction, temporal contiguity also modulates SoA: Higher SoA ratings are observed for effects that appear shortly after the action compared to effects that are delayed, indicating that temporal contiguity enhances SoA (Sato and Yasuda, 2005; Farrer et al., 2013). Other agency cues that contribute to the estimation of SoA are exclusivity, i.e., no one else could have caused the effect, and priority, i.e., action relevant thoughts preceding the action (Wegner and Wheatley, 1999; Wegner and Sparrow, 2004; Vuorre, 2017).

One further factor that has been discussed as an agency cue is the affective valence of the effect (Wilke et al., 2012; Synofzik et al., 2013; Gentsch and Synofzik, 2014). SoA ratings are higher (Beyer et al., 2017; Barlas et al., 2018) and intentional binding is often stronger (Christensen et al., 2016; Yoshie and Haggard, 2017; but see Moreton et al., 2017; Barlas et al., 2018) for positive compared to negative effects. For instance, intentional binding is stronger for effects associated with monetary reward (which is thought to induce positive emotions) than monetary loss (which is thought to induce negative emotions, Takahata et al., 2012) and for positive emotional sounds (e.g., cheers, laughs) than for negative emotional sounds (e.g., screams, retches) as effects (Yoshie and Haggard, 2013). Likewise, people are more likely to attribute positive effects to themselves than negative effects (Gentsch et al., 2015). One explanation for such results is that the self-serving bias, a cognitive distortion of reality, influences SoA (Takahata et al., 2012; Barlas and Obhi, 2014; Christensen et al., 2016). In order to enhance ones self-worth or self-esteem (for an overview see Shepperd et al., 2008) people tend to attribute positive outcomes internally to one’s own actions, own skills, personal traits, character, or efforts, whereas negative outcomes are attributed externally to actions of others or situational circumstances (Bradley, 1978; Greenberg et al., 1982; Shepperd et al., 2008).

Even though it has been shown that people use different agency cues to estimate their SoA over actions or effects, the question arises whether agency cues are used similarly across different cultures. Western and Eastern cultures differ in self-related constructs and construals of the self (Markus and Kitayama, 1991; Heine, 2001). Those differences may extend to conceptions of SoA over actions and effects (Frith, 2013; Voyer and Franks, 2014). Western cultures are more individualistic and characterized by the pursuit of individual goals, behavior based on personal attitudes, and an independent self (Hui and Triandis, 1986; Triandis, 2001). In contrast, Eastern cultures are more collectivistic and characterized by the pursuit of in-group goals, behavior based on in-group norms, and a self, which is interdependent with other members of the group (Hui and Triandis, 1986; Triandis, 2001). Those differences also extend to information processing strategies (Nisbett and Miyamoto, 2005). Holistic information processing with a focus on relationships and similarities between objects as well as the context in which an object is located is more common in Eastern cultures, whereas analytic information processing with a focus on salient objects independent from the context is more common in Western cultures (Nisbett and Miyamoto, 2005). Additionally, cultures differ in their processing and expression of emotions (Kitayama et al., 2000). Ego-focused emotions (e.g., anger, pride), which have the individuals’ internal state like own desires and the individual’s own goals as primary referents, are more frequently expressed and experienced in Western cultures, whereas other-focused emotions (e.g., sympathy), which have another person as primary referent, are more frequently experienced in Eastern cultures (Kitayama et al., 2000, 2006). Further, Eastern cultures are more likely than Western cultures to incorporate information from the social context into the processing of emotions (Masuda et al., 2008).

One may expect that those differences in self-construal, cognition, and emotion also affect how people experience their actions and effects and how they infer SoA (Frith, 2013). Accordingly, depending on culture, people have different models of SoA. People in Western cultures often have disjoint models of SoA, in which actions are independent from others, freely chosen according to one’s own intentions and goals, and in which individuals are fully responsible for the effects of their actions (Markus and Kitayama, 2003). People in Eastern cultures often have conjoint models of SoA, in which actions are interdependent with others, chosen in accordance with interpersonal intentions and expectations of others, and in which responsibility for the effects of actions is shared (Markus and Kitayama, 2003).

So far only a few studies have systematically investigated the use of agency cues in different cultures. In many ways, agency cues seem to be processed similarly in Western and Eastern cultures, but some subtle differences between cultures are also observed. In both Western and Eastern cultures SoA ratings are higher if the effect of an action is previously primed compared to when it is not. Thus, a match between expected and actual effects seems to enhance SoA independent of culture, indicating that a universal component of SoA exists (Aarts et al., 2010). However, Aarts et al. (2010) observed that SoA ratings were higher in Western than Eastern cultures. Barlas and Obhi (2014) observed higher SoA ratings for consonant chords (pleasant effects) than for dissonant ones (unpleasant effects) in both Western participants and Non-Western immigrants living in a Western country. However, intentional binding was only enhanced in Western participants. Barlas and Obhi (2014) suggested that enhanced intentional binding in Western participants may be either explained by a stronger self-serving bias in Western cultures or by higher familiarity with/more frequent exposure to consonant than dissonant chords in Western participants.

In the present study, we investigated cultural differences in the use of different agency cues. We focused on action-effect congruency and temporal relation between action and effect as agency cues as those have been rarely investigated cross-culturally. We further focused on affective valence of the effect as agency cue, because it has been claimed that the affective valence of an effect modulates SoA differently in Eastern and Western participants (Barlas and Obhi, 2014). In the present study, Western (Austrian) and Eastern (Mongolian) participants living in the respective country were investigated. Participants first learned action-effect associations between keypresses and affectively valenced stimuli (happy and sad smileys, see Ganster et al., 2012; Moreton et al., 2017). Afterward, participants performed the same keypresses. Keypresses were followed again by happy and sad smileys that could be either congruent or incongruent with the previously acquired action-effect associations. Furthermore, the interval between the keypresses and the smileys was manipulated. In each trial participants were asked to rate how likely the effect was caused by themselves or by the computer.

We expected that participants of both cultures report higher authorship for congruent than for incongruent effects, because the congruent effects should be predicted and a match between predicted and actual effects enhances SoA ratings (Sato and Yasuda, 2005). Further, we expected higher authorship ratings for effects presented shortly after the action, as this has been observed in previous studies (Sato and Yasuda, 2005; Farrer et al., 2013). Moreover, participants should report higher authorship for positive than for negative effects (cf. Barlas and Obhi, 2014). One question of the present study was whether affective valence will be affected by culture. Barlas and Obhi (2014) observed cross-cultural differences regarding affective valence only in indirect, but not in direct measures of SoA. However, there are reasons to assume that in the present task culture might influence authorship ratings. Barlas and Obhi (2014) compared Western participants with Non-Western immigrants living in a Western country. Therefore, two conflicting belief systems in Non-Western participants (one explicit system promoted in the new country and one implicit system, which is still in accordance with the belief-system of their home country), might have caused the differences in direct/indirect measures in their study (Hetts et al., 1999; Barlas and Obhi, 2014). We speculate that we might observe a higher difference in authorship ratings between positive and negative effects in Western participants than in Eastern participants for two reasons. First, the present task is an ego-focused task, in which participants act individually, no social exchange occurs, and affectively valenced stimuli are presented without a social context. Eastern participants might pay less attention to the affective valence of the effect, because when processing emotions Eastern cultures depend more on information from the social context in which they occur than Western cultures (Masuda et al., 2008). Second, the affective valence of the effect may have less influence on Eastern participants’ SoA, because the self-serving bias (i.e., the tendency to attribute positive outcomes to one’s own actions and negative outcomes to the action of others) is less pronounced in Eastern than in Western cultures (Heine and Hamamura, 2007; see Mezulis et al., 2004 for a meta-analysis).

## Materials and Methods

### Participants

Originally 181 students (77 Austrians, 104 Mongolians) took part in the study. Forty eight participants were excluded from analysis for reasons stated below. The final sample consisted of 72 Austrian (sex: 53 female, 19 male; handedness: 64 right, 8 left; age in years: *M* = 20.6, *SD* = 1.3) university students of the University for Health Sciences, Medical Informatics and Technology in Austria (UMIT) and 61 Mongolian (sex: 44 female, 17 male; handedness: 52 right, 3 left, 6 ambidextrous; age in years: *M* = 19.9, *SD* = 1.3) university students of the National University of Mongolia (NUM). The study was carried out in accordance with the recommendations of the Research Committee for Scientific and Ethical Questions (RCSEQ) in Tyrol, Austria. The protocol was approved by the RCSEQ. All subjects gave written informed consent.

The minimum sample size for all effects of interest (i.e., all main effects and the interaction between culture and valence) was estimated using G^∗^Power (version 3.1.9.2). We assumed a medium effect size (*f* = 0.2) and medium correlations (*r* = 0.5) between all conditions. Further, alpha was set at 0.05 and the power (1-beta) at 0.9. This resulted in an estimated sample size of 68 (34 participants per group).

### Material and Procedure

Participants were tested in groups of maximal 30 participants in the computer labs of the respective university. Participants were seated at desks approximately 50 cm in front of a computer screen. For Austrian participants HP z23i monitors (screen: 23″, vertical refresh rate: 60 Hz, resolution: 1920 × 1080 pixels) and for Mongolian participants Intel i3 monitors (screen: 19″, vertical refresh rate: 60 Hz, resolution: 1366 × 768 pixels) were used. The experiment was programmed using SR Research Experimental Builder version 1.10.1630^1^. Keypresses were performed on the number keys 6 and 8 on a keyboard (response keys) using the ring finger and index finger of the left hand.

**FIGURE 1:**
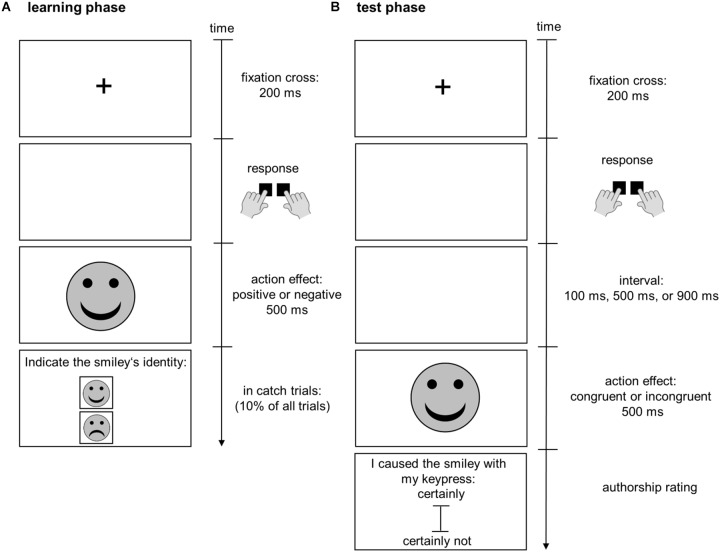
Depiction of the trial procedures in the learning phase **(A)** and test phase **(B)**. In the learning phase, participants learned to associate actions (keypresses) with positive or negative effects (smileys). In the learning phase, participants freely chose to press one of two keys. Keypresses were immediately and consistently followed by happy or sad smileys. Ten percent of trials were catch trials in which participants were asked to indicate the identity of the just presented smiley at the end of the trial. In the test phase, participants performed the same keypresses. After different intervals positive and negative effects, which were either congruent or incongruent with the previously acquired action-effect associations, were presented. Participants were asked to rate how likely the effect was caused by themselves.

In the first phase of the experiment, participants learned action-effect associations (learning phase, see Figure 1, panel A). A trial started with the presentation of a black fixation cross (0.9 cm × 0.9 cm) on a white background in the center of the screen for 200 ms. Participants were asked not to respond immediately to the disappearance of the fixation cross, but to press one of the two response keys at a time of their choice after the disappearance of the fixation cross. We chose free-choice action selection because free-choice may promote the acquisition of action-effect associations (Herwig et al., 2007; Herwig and Waszak, 2009) or the subsequent use of action-effect associations (Pfister et al., 2011; Sommer and Lukas, 2018) more than forced-choice. Participants were instructed to try to press each response key equally often, but to avoid using a regular pattern of keypresses. Immediately (0 ms) after the keypress an affectively valenced effect (happy or sad smiley; color: gray, diameter: 5 cm) was presented in the center of the screen for 500 ms. We chose smileys as effects, because we assumed that students from both cultures are familiar with using them to express affective states due to the use of digital media. The key-smiley assignment was counterbalanced across participants. Participants were not informed about the key-smiley assignment prior to the learning phase. After an inter-trial-interval of 1000 ms the next trial started. To ensure that participants paid attention to the smileys, 10% of the trials were catch trials, in which they were asked to indicate the smiley’s identity 500 ms after its presentation. Participants were asked to indicate the smiley’s identity by choosing via mouse click between a simultaneously presented happy and sad smiley (diameter: 2.3 cm). Those smileys were presented above each other, each in a separate square (3 cm × 3 cm, distance between the squares: 2 cm) in the upper half of the screen. The learning phase consisted of 120 trials.

After the learning phase, the test phase commenced (see Figure 1, panel B). The trial procedure was similar to the learning phase apart from the following differences: After the keypresses, smileys were not presented immediately but after a variable interval (100, 500, or 900 ms). In one-half of the trials the presented smiley was congruent with the key-smiley assignment in the learning phase. In the other half of the trials the presented smiley was incongruent with the key-smiley assignment in the learning phase. Participants were informed that the occurrence of the smiley on the screen was either caused by their keypress or was automatically generated by the computer independent of their keypress. In every trial 500 ms after the disappearance of the smiley participants were asked to rate their authorship over the smiley by indicating via mouse click on a vertically aligned visual analog scale (14.5 cm) from “certainly not” to “certainly” how sure they are that they caused the smiley with their keypress. After an inter-trial-interval (1000 ms) the next trial started. In contrast to the learning phase no catch trials were presented. Each interval was presented 40 times (20 congruent and 20 incongruent trials) in random order. In total the test phase consisted of 120 trials.

### Data Analysis

If participants paid only little attention to the effects of their actions in the learning phase, learning of action-effect associations might be reduced or prevented. Thus, participants were excluded from analysis if they answered less than 75% of the catch trials (i.e., the question about the identity of the presented smiley) correctly (*N* = 26). Further, participants were excluded from analysis if the percentage ratio between the two keypresses was higher than 70:30 in the learning phase (*N* = 14), because if participants did not adhere to instructions and produced an unbalanced number of keypresses learning of one action-effect association might be less pronounced than learning of the other. The 70:30 ratio was chosen because visual inspection of the data indicated that the majority of participants had a ratio more balanced than this and because the probability of having a keypress ratio higher than that is highly unlikely if one assumes keypresses are random (*p* < 0.001). In the test phase, an unbalanced number of trials per condition results in unreliable measures. Thus, participants were further excluded from analysis if the percentage ratio between the two keypresses was higher than 70:30 in the test phase (*N* = 8).

The lowest score of the authorship rating was defined as 0 and the highest score as 100. Data from two of the participants included outliers with authorship ratings three standard deviations above or below the mean of the other participants in the group. However, because the pattern of results was the same with and without those participants, we report the results for all participants.

An ANOVA with the between-participants factor culture (Austria, Mongolia) and the within-participants factors valence (positive, negative), congruency (congruent, incongruent), and interval (100, 500, and 900 ms) was performed on participants’ authorship ratings. If Mauchly’s test indicated that the assumption of sphericity was violated, Greenhouse-Geisser corrected *F*-values, *p*-values, and Greenhouse-Geisser’𝜀 are reported. *Post hoc* comparisons were conducted using paired *t*-tests. Significance values were adjusted for multiple testing using Sidak correction. When several *post hoc* comparisons are reported together, minimum (*p*_min_) or maximum *p*-values (*p*_max_) are reported.

## Results

**FIGURE 2:**
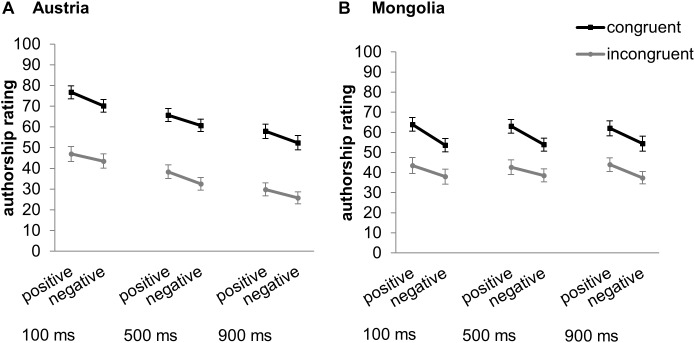
Means and standard errors of authorship ratings depending on valence (positive, negative), congruency (congruent, incongruent), and interval (100, 500, and 900 ms), separately for Austrian **(A)** and Mongolian **(B)** participants.

Means and standard errors of authorship ratings separately for the Austrian and Mongolian participants are depicted in Figure 2. A significant main effect of valence, *F*(1,131) = 41.99, *p* < 0.001, $\eta_{p}^{2}$ = 0.24, indicated higher authorship ratings for positive (*M* = 52.88, *SD* = 17.04) than for negative (*M* = 46.82, *SD* = 16.01) effects over all congruency conditions and over all intervals. A significant main effect of congruency, *F*(1,131) = 49.22, *p* < 0.001, $\eta_{p}^{2}$ = 0.27, indicated higher authorship ratings for congruent (*M* = 61.44, *SD* = 23.8) than for incongruent (*M* = 38.25, *SD* = 24.99) effects over all valence conditions and over all intervals. Additionally, a significant interaction between congruency and valence, *F*(1,131) = 4.44, *p* = 0.037, $\eta_{p}^{2}$ = 0.033, indicated a significantly higher difference in authorship ratings between positive and negative effects with congruent (positive: *M* = 65.06, *SD* = 25.27; negative: *M* = 57.82, *SD* = 24.3; difference: *M* = 7.24, *SD* = 13.92) than incongruent effects (positive: *M* = 40.69, *SD* = 26.9; negative: *M* = 35.81, *SD* = 24.35; difference: *M* = 4.88, *SD* = 11.61; *p* = 0.045). The significant main effect of interval, *F*(2,262) = 24.45, *p* < 0.001, $\eta_{p}^{2}$ = 0.16, 𝜀 = 0.61, could not be interpreted due to a significant interaction between interval and culture, *F*(2, 262) = 22.71, *p* < 0.001, $\eta_{p}^{2}$ = 0.15, 𝜀 = 0.61. In Austrian participants significantly higher authorship ratings after the 100 ms interval than after the 500 ms and 900 ms interval (*p*_max_ < 0.001), and significantly higher authorship ratings after the 500 ms than after the 900 ms interval (*p* < 0.001) were observed. In Mongolian participants no significant differences between intervals were observed (*p*_min_ = 0.99). Further, at the 100 ms interval authorship ratings were significantly higher in Austrians than in Mongolians (*p* = 0.005), at the 500 ms interval authorship ratings did not significantly differ between cultures (*p* = 0.95), and at the 900 ms interval authorship ratings were significantly lower in Austrians than in Mongolians (*p* = 0.011). Neither the main effect of culture [*F*(1,131) = 0.03, *p* = 0.86, $\eta_{p}^{2}$ < 0.001] nor any of the remaining interactions were significant [congruency × culture: *F*(1,131) = 2.36, *p* = 0.13, $\eta_{p}^{2}$ = 0.018, valence × culture: *F*(1,131) = 1.25, *p* = 0.27, $\eta_{p}^{2}$ = 0.009, interval × congruency: *F*(2,262) = 0.16, *p* = 0.84, $\eta_{p}^{2}$ = 0.001, 𝜀 = 0.94, interval × congruency × culture: *F*(2,262) = 0.011, *p* = 0.99, $\eta_{p}^{2}$ < 0.001, 𝜀 = 0.94, interval × valence: *F*(2,262) = 0.17, *p* = 0.84, $\eta_{p}^{2}$ = 0.001, interval × valence × culture: *F*(2,262) = 0.41, *p* = 0.67, $\eta_{p}^{2}$ = 0.003, congruency × valence × culture: *F*(1,131) = 1.08, *p* = 0.3, $\eta_{p}^{2}$ = 0.008, interval × congruency × valence: *F*(2,262) = 1.02, *p* = 0.36, $\eta_{p}^{2}$ = 0.008, interval × congruency × valence x culture: *F*(2,262) = 1.38, *p* = 0.25, $\eta_{p}^{2}$ = 0.01].

## Discussion

The aim of the present study was to examine to what extent the use of different agency cues (action-effect congruency, temporal relation between action and effect, and affective valence of the effect) for SoA judgments differs between Eastern and Western cultures. Students from Austria and Mongolia performed keypresses, which were followed by positive or negative effects (happy or sad smileys) during a learning phase. Afterward, participants performed the same keypresses, which were, after different intervals, followed by positive or negative effects. The effects were either congruent or incongruent with the previously acquired action-effect associations. In each trial participants were asked to rate their authorship over the presented effect. Higher authorship ratings were observed for congruent than for incongruent effects and for positive than for negative effects. The difference between positive and negative effects was higher with congruent effects than incongruent effects. However, no significant difference regarding the affective valence of the effects was observed between Austrian and Mongolian participants. Interestingly, authorship ratings decreased with increasing interval in Austrian participants, but not in Mongolian participants.

Agency cues like a match between predicted and actual effects of an action enhance SoA (Frith et al., 2000; Blakemore et al., 2002). Correspondingly, we observed higher authorship ratings for congruent than for incongruent effects in both cultures. This shows that congruency between action and effect enhances SoA and confirms that predictive processes about an effect’s identity play a role for SoA. The effect is in line with previous studies, which observed also higher SoA ratings for congruent compared to incongruent effects (Sato and Yasuda, 2005; Spengler et al., 2009) and corresponds to Aarts et al. (2010), who observed higher SoA ratings in both Western and Eastern cultures when actual effects corresponded to expected effects. Thus, congruency between action and effect might be a central universal agency cue, on which people from different cultures rely when asked to judge their SoA over events.

In correspondence with previous studies we observed higher authorship ratings for positive compared to negative effects. Thus, our results support the view that SoA is modulated by the affective valence of effects (Synofzik et al., 2013; Barlas and Obhi, 2014; Gentsch and Synofzik, 2014; Beyer et al., 2017; Barlas et al., 2018). Further, our results are in line with Barlas and Obhi (2014), who observed higher SoA ratings for positive than for negative effects in both, Western and Non-Western participants. Thus, affective valence is used as universal agency cue. We had speculated that we might find a higher difference in authorship ratings between positive and negative effects in Western participants than in Eastern participants. Several explanations can be brought forward that we did not observe the expected effect.

First, in accordance with the results of Barlas and Obhi (2014), direct measures of SoA might not be as sensitive to cross-cultural differences as indirect measures. However, the direct/indirect dissociation in the study of Barlas and Obhi (2014) was probably observed because they investigated Non-Western immigrants living in a Western country. Those participants may have two conflicting belief systems (one explicit system promoted in the new country and one implicit system, which is still in accordance with the belief-system of their home country) (Hetts et al., 1999; Barlas and Obhi, 2014). In the present study, Mongolians living in Mongolia were investigated. We therefore think it is unlikely that the use of a direct measure instead of an indirect measure might be the reason that we did not find significant cultural differences in the use of affective valence as an agency cue.

A second reason might be that our methods (i.e., the task and the testing situation) were not suited to evoke the crucial cultural concepts. The influence of affective valence on SoA is frequently explained by the self-serving bias, i.e., the tendency to attribute positive outcomes to one’s own actions and negative outcomes to the action of others (Takahata et al., 2012; Yoshie and Haggard, 2013; Barlas and Obhi, 2014). Previously, a higher self-serving bias has been observed in Western than in Eastern participants (Mezulis et al., 2004). However, people are more likely to attribute negative consequences to themselves (low self-serving bias) when the perceived probability of improvement is high (Duval and Silvia, 2002). In the current task there was no way to improve the outcome, i.e., to avoid negative effects. Therefore participants from both cultures might have equally felt less authorship over negative than positive effects. Further, due to the group testing situation participants may have felt a high degree of anonymity. When anonymity and confidentiality are strongly emphasized, one of the reasons for a low self-serving bias, namely to present oneself as modest (Kurman, 2003), does not apply anymore. This leads to an observable self-serving bias even in Eastern cultures (Kudo and Numazaki, 2003).

A third reason might be that the studied populations did not differ in the crucial cultural concepts. We investigated students. Although they are from different cultural backgrounds, students may have a lot in common in terms of achievement motivation, independence, intellectual interests, and academic goals. Those commonalities might extend to the self-related constructs which influence the use of affective valence of an effect as agency cue.

In Austrian participants authorship ratings decreased with increasing interval. The decrease of authorship ratings with increasing interval might be due to temporal contiguity and/or temporal prediction. Temporal contiguity refers to the temporal proximity between action and effect. High temporal contiguity increases SoA ratings (Sato and Yasuda, 2005; Farrer et al., 2013). Temporal prediction refers to expectations about the point in time at which an effect will occur (Haering and Kiesel, 2015). Based on previous experiences about usual action-effect intervals, predictions concerning the timing of certain effects are formed (Haering and Kiesel, 2012; Walsh and Haggard, 2013). In case temporal predictions match the actual timing of effects SoA ratings increase (Haering and Kiesel, 2015). In the present study, temporal contiguity and temporal prediction cannot be dissociated. At the shortest interval temporal contiguity was high. Further, at the shortest interval the predicted timing (in the learning phase effects were presented without delay, so participants may predict the effect to occur immediately) corresponded most to the actual timing (100 ms delay). Whatever is the case, our results suggest that information regarding the temporal occurrence of the effect is used as an agency cue in Austrian participants.

Surprisingly, authorship ratings did not significantly differ between intervals in Mongolian participants. In line with this, authorship ratings were significantly lower in Mongolians than in Austrians at the 100 ms interval, did not significantly differ between cultures at the 500 ms interval, and were higher in Mongolians than in Austrians at the 900 ms interval. Those results indicate that temporal cues might not be used to estimate SoA in Mongolians. One reason for this may be that cross-cultural differences exist in the conception of time (Levine, 2006). The pace of life and correspondingly concepts of time seem to differ between Eastern and Western or collectivistic and individualistic cultures, respectively (Block et al., 1996; Brislin and Kim, 2003; Levine, 2006). Whereas in Western/individualistic cultures time-efficiency and punctuality are important and people are anxious not to waste their time, this is not so much the case in Eastern/collectivistic cultures (Brislin and Kim, 2003). Further, many Western/individualistic cultures experience time as linear, moving in one direction from the past to the present to the future, whereas many Eastern/collectivistic cultures have cyclical concepts of time, in which repetition or reoccurrence of events like the cycle of day and night or the seasonal cycle are emphasized (Dahl,, 1995; Levine, 2006; Widlok, 2014). Cyclical time concepts might result in different concepts of causality (Widlok, 2014). Perceived causality is closely related to SoA (De Vignemont and Fourneret, 2004) and usually arises if the temporal contiguity between two events is high. One may speculate that cyclical time concepts might result in less concern with or less attention to the temporal chronology of events when causal relationships are inferred (Widlok, 2014). It is also possible that temporal information processing is different depending on the underlying time concept. Thus, temporal chronology of events or timing of events might not be a reliable agency cue in Eastern people, which led to equally high authorship ratings for all three intervals in our experiment.

With reference to the cue integration view (Synofzik et al., 2009; Moore and Fletcher, 2012), our results indicate that different agency cues like action-effect congruency, affective valence of effects, and in Western participants also the temporal relation between action and effect are used to estimate one’s SoA. Our results indicate that SoA increases with the number of cues indicating that oneself caused the action. Interestingly, we observed a higher difference in authorship ratings between positive and negative effects with congruent than with incongruent effects. This indicates an overadditive effect of congruency and affective valence, which might reflect the conjoint working of cues. Cue integration models of SoA, do not only assume that different agency cues are used, but also that they are integrated to estimate one’s SoA (Synofzik et al., 2009; Moore and Fletcher, 2012). Such an integration seems to take place between congruency and affective valence. Surprisingly, no interplay between congruency and interval was observed, which stands in contrasts to the results of Sato and Yasuda (2005), who in some experiments only observed an effect of congruency for short intervals. Such findings indicate that if the interval between action and effect is too long one might not perceive oneself as the cause of the effect, resulting in reduced SoA ratings even for congruent effects. Our intervals were longer than those used in Sato and Yasuda (2005). However, we used visual stimuli and not auditory stimuli. The intervals between actions and effects, which are suitable to evoke SoA, might differ between effect modalities. Thus, for the visual effects used in the present study the intervals might not have been long enough to reduce SoA to the extent that the effect of congruency disappeared.

A limitation of the present study is that we do not know whether positive effects enhanced SoA, whether negative effects weakened SoA, or whether both was the case, because we had no comparison condition with neutral effects. A further limitation is the use of student samples, which are not representative for the whole population of a country. Students might be a special group as they may differ in values, norms, and self-related concepts from the rest of the population. A final limitation is that we did not use additional measures to investigate whether participants from both cultures differed in cultural concepts which might be related to SoA.

Further studies might investigate the use of agency cues in different subsamples of the population (e.g., rural vs. urban, high-educated vs. low-educated) in Western and Eastern cultures. Further, even though many Eastern cultures share some cultural values and norms, there is still a wide variety of cultural differences between countries (the same holds true for Western cultures). Accordingly, one may not necessarily generalize findings obtained with Mongolian and Austrian participants to other countries. Further research should investigate the role of agency cues in different Eastern and Western cultures.

## Conclusion

In conclusion, our results support the view that different agency cues are used to estimated one’s SoA. Further, whereas some agency cues like congruency between action and effect and affective valence of effects might be used to infer SoA regardless of culture, the use of others like temporal cues might vary depending on culture.

## Data Availability

Datasets are available on request. The raw data supporting the conclusions of the manuscript will be made available by the authors, without undue reservation, to any qualified researcher.

## Author Contributions

VB contributed to designing the research, programmed the experiments, supervised the data collection in Austria, contributed to analyzing the data, and wrote the first draft of the manuscript. MR contributed to designing the research, analyzing the data, and writing the manuscript. DW contributed to designing the research and writing the manuscript. KB, ES, and TM contributed to supervising the data collection in Mongolia and gave feedback on the manuscript.

## Conflict of Interest Statement

The authors declare that the research was conducted in the absence of any commercial or financial relationships that could be construed as a potential conflict of interest.

## Footnotes

1 http://www.sr-research.com/eb

## References

[B1] Aarts H. Oikawa M. Oikawa H. (2010). Cultural and universal routes to authorship ascription: effects of outcome priming on experienced self-agency in the Netherlands and Japan. *J. Cross-Cult. Psychol.* 41 87–98. 10.1177/0022022109349511

[B2] Aarts H. van den Bos K. (2011). On the foundations of beliefs in free will intentional binding and unconscious priming in self-agency. *Psychol. Sci.* 22 532–537. 10.1177/0956797611399294 21317370

[B3] Barlas Z. Hockley W. E. Obhi S. S. (2018). Effects of free choice and outcome valence on the sense of agency: evidence from measures of intentional binding and feelings of control. *Exp. Brain Res.* 236 129–139. 10.1007/s00221-017-5112-3 29080100

[B4] Barlas Z. Obhi S. S. (2014). Cultural background influences implicit but not explicit sense of agency for the production of musical tones. *Conscious. Cognit.* 28 94–103. 10.1016/j.concog.2014.06.013 25051499

[B5] Berberian B. Sarrazin J.-C. Le Blaye P. Haggard P. (2012). Automation technology and sense of control: a window on human agency. *PLoS One* 7:e34075. 10.1371/journal.pone.0034075 22479528 PMC3316600

[B6] Beyer F. Sidarus N. Bonicalzi S. Haggard P. (2017). Beyond self-serving bias: diffusion of responsibility reduces sense of agency and outcome monitoring. *Soc. Cogn. Affect. Neurosci.* 12 138–145. 10.1093/scan/nsw160 27803288 PMC5390744

[B7] Blakemore S. J. Wolpert D. M. Frith C. D. (2002). Abnormalities in the awareness of action. *Trends Cognit. Sci.* 6 237–242. 10.1016/S1364-6613(02)01907-1 12039604

[B8] Block R. A. Buggie S. E. Matsui F. (1996). Beliefs about time: cross-cultural comparisons. *J. Psychol.* 130 5–22. 10.1080/00223980.1996.9914984

[B9] Bradley G. W. (1978). Self-serving biases in the attribution process: a reexamination of the fact or fiction question. *J. Pers. Soc. Psychol.* 36 56–71. 10.1037/0022-3514.36.1.56

[B10] Brislin R. W. Kim E. S. (2003). Cultural diversity in people’s understanding and uses of time. *Appl. Psychol.* 52 363–382. 10.1111/1464-0597.00140

[B11] Christensen J. F. Yoshie M. Di Costa S. Haggard P. (2016). Emotional valence, sense of agency and responsibility: a study using intentional binding. *Conscious. Cognit.* 43 1–10. 10.1016/j.concog.2016.02.016 27174794

[B12] Dahl Ø. (1995). When the future comes from behind: malagasy and other time concepts and some consequences for communication. *Int. J. Intercult. Relat.* 19 197–209. 10.1016/0147-1767(95)00004-U

[B13] De Vignemont F. Fourneret P. (2004). The sense of agency: a philosophical and empirical review of the “Who” system. *Conscious. Cognit.* 13 1–19. 10.1016/S1053-8100(03)00022-9 14990237

[B14] Dewey J. A. Knoblich G. (2014). Do implicit and explicit measures of the sense of agency measure the same thing? *PLoS One* 9:e110118. 10.1371/journal.pone.0110118 25330184 PMC4199671

[B15] Duval T. S. Silvia P. J. (2002). Self-awareness, probability of improvement, and the self-serving bias. *J. Pers. Soc. Psychol.* 82 49–61. 10.1037/0022-3514.82.1.49 11811633

[B16] Ebert J. P. Wegner D. M. (2010). Time warp: authorship shapes the perceived timing of actions and events. *Conscious. Cognit.* 19 481–489. 10.1016/j.concog.2009.10.002 19896868 PMC2836403

[B17] Farrer C. Valentin G. Hupé J. M. (2013). The time windows of the sense of agency. *Conscious. Cognit.* 22 1431–1441. 10.1016/j.concog.2013.09.010 24161792

[B18] Frith C. (2013). The psychology of volition. *Exp. Brain Res.* 229 289–299. 10.1007/s00221-013-3407-6 23354664 PMC3745827

[B19] Frith C. D. Blakemore S. J. Wolpert D. M. (2000). Abnormalities in the awareness and control of action. *Philos. Trans. R. Soc. Lon. B* 355 1771–1788. 10.1098/rstb.2000.0734 11205340 PMC1692910

[B20] Ganster T. Eimler S. C. Krämer N. C. (2012). Same same but different!? The differential influence of smilies and emoticons on person perception. *Cyberpsychol. Behav. Soc. Netw.* 15 226–230. 10.1089/cyber.2011.0179 22394421

[B21] Gentsch A. Synofzik M. (2014). Affective coding: the emotional dimension of agency. *Front. Hum. Neurosci.* 8:608. 10.3389/fnhum.2014.00608 25161616 PMC4130111

[B22] Gentsch A. Weiss C. Spengler S. Synofzik M. Schütz-Bosbach S. (2015). Doing good or bad: how interactions between action and emotion expectations shape the sense of agency. *Soc. Neurosci.* 10 418–430. 10.1080/17470919.2015.1006374 25644692

[B23] Greenberg J. Pyszczynski T. Solomon S. (1982). The self-serving attributional bias: beyond self-presentation. *J. Exp. Soc. Psychol.* 18 56–67. 10.1016/0022-1031(82)90081-6

[B24] Haering C. Kiesel A. (2012). Time in action contexts: learning when an action effect occurs. *Psychol. Res.* 76 336–344. 10.1007/s00426-011-0341-8 21584676

[B25] Haering C. Kiesel A. (2015). Was it me when it happened too early? Experience of delayed effects shapes sense of agency. *Cognition* 136 38–42. 10.1016/j.cognition.2014.11.012 25490127

[B26] Haggard P. (2017). Sense of agency in the human brain. *Nat. Rev. Neurosci.* 18 196–207. 10.1038/nrn.2017.14 28251993

[B27] Haggard P. Clark S. Kalogeras J. (2002). Voluntary action and conscious awareness. *Nat. Neurosci.* 5 382–385. 10.1038/nn827 11896397

[B28] Haggard P. Tsakiris M. (2009). The experience of agency: feelings, judgments, and responsibility. *Curr. Direct. Psychol. Sci.* 18 242–246. 10.1111/j.1467-8721.2009.01644.x

[B29] Heine S. J. (2001). Self as a cultural product. An examination of east Asian and north American selves. *J. Pers.* 69 881–906. 10.1111/1467-6494.696168 11767822

[B30] Heine S. J. Hamamura T. (2007). In search of East Asian self-enhancement. *Personal. Soc. Psychol. Rev.* 11 4–27. 10.1177/1088868306294587 18453453

[B31] Herwig A. Prinz W. Waszak F. (2007). Two modes of sensorimotor integration in intention-based and stimulus-based actions. *Quart. J. Exp. Psychol.* 60 1540–1554. 10.1080/17470210601119134 17853217

[B32] Herwig A. Waszak F. (2009). Short article: intention and attention in ideomotor learning. *Quart. J. Exp. Psychol.* 62 219–227. 10.1080/17470210802373290 18932052

[B33] Hetts J. J. Sakuma M. Pelham B. W. (1999). Two roads to positive regard: implicit and explicit self-evaluation and culture. *J. Exp. Soc. Psychol.* 35 512–559. 10.1006/jesp.1999.1391

[B34] Hui C. H. Triandis H. C. (1986). Individualism-collectivism a study of cross-cultural researchers. *J. Cross-Cult. Psychol.* 17 225–248. 10.1177/0022002186017002006

[B35] Kitayama S. Markus H. R. Kurokawa M. (2000). Culture, emotion, and well-being: good feelings in Japan and the United States. *Cognit. Emot.* 14 93–124. 10.1080/026999300379003

[B36] Kitayama S. Mesquita B. Karasawa M. (2006). Cultural affordances and emotional experience: socially engaging and disengaging emotions in Japan and the United States. *J. Pers. Soc. Psychol.* 91 890–903. 10.1037/0022-3514.91.5.890 17059308

[B37] Kudo E. Numazaki M. (2003). Explicit and direct self-serving bias in Japan: reexamination of self-serving bias for success and failure. *J. Cross-Cult. Psychol.* 34 511–521. 10.1177/0022022103256475

[B38] Kurman J. (2003). Why is self-enhancement low in certain collectivist cultures? An investigation of two competing explanations. *J. Cross-Cult. Psychol.* 34 496–510. 10.1177/0022022103256474

[B39] Levine R. N. (2006). *A Geography of Time: The Temporal Misadventures of a Social Psychologist, or How Every Culture Keeps Time Just a Little Bit Differently*. Oxford: Oneworld Publications.

[B40] Markus H. R. Kitayama S. (1991). Culture and the self: implications for cognition, emotion, and motivation. *Psychol. Rev.* 98 224–253. 10.1037/0033-295X.98.2.224

[B41] Markus H. R. Kitayama S. (2003). “Models of agency: sociocultural diversity in the construction of action,” in *Cross-Cultural Differences in Perspectives on the Self*, eds Murphy-Berman V. Berman J. J. (Lincoln: University of Nebraska Press), 1–58. 14569670

[B42] Masuda T. Ellsworth P. C. Mesquita B. Leu J. Tanida S. Van de Veerdonk E. (2008). Placing the face in context: cultural differences in the perception of facial emotion. *J. Pers. Soc. Psychol.* 94 365–381. 10.1037/0022-3514.94.3.365 18284287

[B43] Mezulis A. H. Abramson L. Y. Hyde J. S. Hankin B. L. (2004). Is there a universal positivity bias in attributions? A meta-analytic review of individual, developmental, and cultural differences in the self-Serving attributional bias. *Psychol. Bull.* 130 711–747. 10.1037/0033-2909.130.5.711 15367078

[B44] Moore J. W. (2016). What is the sense of agency and why does it matter? *Front. Psychol.* 7:1727. 10.3389/fpsyg.2016.01272 27621713 PMC5002400

[B45] Moore J. W. Fletcher P. C. (2012). Sense of agency in health and disease: a review of cue integration approaches. *Conscious. Cognit.* 21 59–68. 10.1016/j.concog.2011.08.010 21920777 PMC3315009

[B46] Moore J. W. Obhi S. S. (2012). Intentional binding and the sense of agency: a review. *Conscious. Cognit.* 21 546–561. 10.1016/j.concog.2011.12.002 22240158

[B47] Moreton J. Callan M. J. Hughes G. (2017). How much does emotional valence of action outcomes affect temporal binding? *Conscious. Cognit.* 49 25–34. 10.1016/j.concog.2016.12.008 28107726

[B48] Nisbett R. E. Miyamoto Y. (2005). The influence of culture: holistic versus analytic perception. *Trends Cognit. Sci.* 9 467–473. 10.1016/j.tics.2005.08.004 16129648

[B49] Pfister R. Kiesel A. Hoffmann J. (2011). Learning at any rate: action-effect learning for stimulus-based actions. *Psychol. Res.* 75 61–65. 10.1007/s00426-010-0288-1 20490862

[B50] Sato A. Yasuda A. (2005). Illusion of sense of self-agency: discrepancy between the predicted and actual sensory consequences of actions modulates the sense of self-agency, but not the sense of self-ownership. *Cognition* 94 241–255. 10.1016/j.cognition.2004.04.003 15617673

[B51] Shepperd J. Malone W. Sweeny K. (2008). Exploring causes of the self-serving bias. *Soc. Pers. Psychol. Compass* 2 895–908. 10.1111/j.1751-9004.2008.00078.x

[B52] Sommer A. Lukas S. (2018). Action-effect associations in voluntary and cued task-switching. *Front. Psychol.* 8:2233. 10.3389/fpsyg.2017.02233 29387027 PMC5776108

[B53] Spengler S. Von Cramon D. Y. Brass M. (2009). Was it me or was it you? How the sense of agency originates from ideomotor learning revealed by fMRI. *Neuroimage* 46 290–298. 10.1016/j.neuroimage.2009.01.047 19457378

[B54] Synofzik M. Vosgerau G. Lindner A. (2009). Me or not me-An optimal integration of agency cues? *Conscious. Cognit.* 18 1065–1068. 10.1016/j.concog.2009.07.007 19683460

[B55] Synofzik M. Vosgerau G. Newen A. (2008). Beyond the comparator model: a multifactorial two-step account of agency. *Conscious. Cognit.* 17 219–239. 10.1016/j.concog.2007.03.010 17482480

[B56] Synofzik M. Vosgerau G. Voss M. (2013). The experience of agency: an interplay between prediction and postdiction. *Front. Psychol.* 4:127. 10.3389/fpsyg.2013.00127 23508565 PMC3597983

[B57] Takahata K. Takahashi H. Maeda T. Umeda S. Suhara T. Mimura M. (2012). It’s not my fault: postdictive modulation of intentional binding by monetary gains and losses. *PLoS One* 7:e53421. 10.1371/journal.pone.0053421 23285293 PMC3532346

[B58] Triandis H. C. (2001). Individualism-collectivism and personality. *J. Pers.* 69 907–924. 10.1111/1467-6494.696169 11767823

[B59] Voyer B. G. Franks B. (2014). Toward a better understanding of self-construal theory: an agency view of the processes of self-construal. *Rev. Gen. Psychol.* 18 101–114. 10.1037/gpr0000003

[B60] Vuorre M. (2017). On time, causation, and the sense of agency. *J. Conscious. Stud.* 24 203–215.

[B61] Walsh E. Haggard P. (2013). Action, prediction and temporal awareness. *Acta Psychol.* 142 220–229. 10.1016/j.actpsy.2012.11.014 23339851

[B62] Wegner D. M. Sparrow B. (2004). “Authorship processing,” in *The Cognitive Neurosciences III*, ed. Gazzaniga M. (Cambridge, MA: MIT Press),1201–1209.

[B63] Wegner D. M. Wheatley T. (1999). Apparent mental causation: sources of the experience of will. *Am. Psychol.* 54 480–492. 10.1037/0003-066X.54.7.480 10424155

[B64] Widlok T. (2014). Agency, time, and causality. *Front. Psychol.* 5:1264. 10.3389/fpsyg.2014.01264 25414683 PMC4222355

[B65] Wilke C. Synofzik M. Lindner A. (2012). The valence of action outcomes modulates the perception of one’s actions. *Conscious. Cognit.* 21 18–29. 10.1016/j.concog.2011.06.004 21757377

[B66] Wolpert D. M. (1997). Computational approaches to motor control. *Trends Cognit. Sci.* 1 209–216. 10.1016/S1364-6613(97)01070-X 21223909

[B67] Wolpert D. M. Flanagan J. R. (2001). Motor prediction. *Curr. Biol.* 11 729–732. 10.1016/S0960-9822(01)00432-8 11566114

[B68] Yoshie M. Haggard P. (2013). Negative emotional outcomes attenuate sense of agency over voluntary actions. *Curr. Biol.* 23 2028–2032. 10.1016/j.cub.2013.08.034 24094850

[B69] Yoshie M. Haggard P. (2017). Effects of emotional valence on sense of agency require a predictive model. *Sci. Rep.* 7:8733. 10.1038/s41598-017-08803-3 28821755 PMC5562802

